# Qualitative immunoassay for the determination of tetracycline antibiotic residues in milk samples followed by a quantitative improved HPLC-DAD method

**DOI:** 10.1038/s41598-022-18886-2

**Published:** 2022-08-25

**Authors:** Moneera N. Alnassrallah, Nourah Z. Alzoman, Aliyah Almomen

**Affiliations:** Department of Pharmaceutical Chemistry, Faculty of Pharmacy, King Saud University, Riyadh, Saudi Arabia

**Keywords:** Environmental sciences, Medical research

## Abstract

Environmental contaminant is one of several problems harming people and wildlife. An example of current emerging contaminants are antibiotics residues that can present in water and food. Although antibiotics are intended to treat or prevent human and animal infections, antibiotics have also been used as animal food supplements for their ability to promote growth and feed efficiency. This overuse of antibacterial has resulted in the accumulation of antibiotics residues in food products which are eventually consumed by human. The continuous unnecessary exposure of human to antibiotics through the direct animals meet or milk, or indirectly through plants or soil can increase the chance of the emergence of multi drug resistance bacteria and consequently adversely affecting human health. New regulations have been imposed regarding antibiotics utilization. Due to the scarce of data regarding antibiotics residue conditions in different types of food intended for human consumption in Saudi Arabia, this study proposed an optimized chromatographic method (HPLC-DAD) followed by an immunoassay approach for specifically detecting tetracyclines antibiotics in animal milk samples. The method was carried out using an RP-C18 column with a mobile phase consisting of 0.01 M KH_2_PO_4_: acetonitrile:methanol (70:20:10, v/v/v) adjusted to pH 4. Improvements were observed in the method in terms of resolution and sensitivity. The protein precipitation method used for extraction demonstrated high percent recoveries of 85–101%. The method was validated according to the guidelines of the International Conference for Harmonization (ICH). It is evidently clear from these findings that the presence of tetracycline and oxytetracycline antibiotics residues in milk products from the Saudi market are below the maximum residual limits (MRLs).

## Introduction

Environmental pollution is a challenge that the world is facing in the current century. This issue is defined as “the contamination of the physical and biological components of the earth/atmosphere system to such an extent that normal environmental processes are adversely affected”^1^. Substances or energy that present above natural levels are considered pollutants^1^. Antibiotics residues are one form of environmental contaminant that can present in animal, plants or soil and can promote the development of microbial resistant. Microbial resistance occurring from the unnecessary use and exposure to antibiotics is defined as changes in bacterial resistance mechanisms or the development of new mechanisms in response to specific antibiotics, a classes of antibiotics, or several types of antibiotic to whatso-called multidrug-resistant pathogens (MDR)^2^. In attempt to preserve antimicrobial activity, regulatory authorities have restricted the prescription process of antibiotics. However, antibiotics are also used as food preservatives, to promote the growth, and to enhance the productivity of cattle and poultry in addition to their use in veterinary medicine^3–5^. Prior research has demonstrated that the use of high levels of antibiotics to increase animal productivity is associated with the presence of antibiotics residues in animal-based foods^6–8^. The continuous exposure of human to antibiotics residues can be harmful to human increasing the possibility of developing allergic reactions, disrupting normal intestinal flora, or can transfer antibiotic-resistant bacteria (ARB) or antibiotics resistance genes (ARGs) from animals to humans^5^.

Tetracycline (TTR), chlortetracycline (CTC) , and oxytetracycline (OXY), are among the most commonly used antibiotics in animal farming due to their effectiveness and low cost^9,10^. Many studies have demonstrated the inappropriate utilization of these antibiotics worldwide and the consequences of such practice^6,11^. For example, there is uncontrolled use of antibiotics in the mixed feed of animals or as preservatives in the food industry^12,13^. Additionally, antibiotics are often fed to animals directly before slaughter or introduced into the carotid artery immediately after slaughter to increase the storage period of fresh meat^12^. Eventually, the residual antibiotic concentrations may exceed the maximum residual limits (MRLs) permitted by the European Union (EU) and World Health Organization (WHO).

Tetracycline antibiotics (TCs) determination methods have been extensively investigated by many analytical methods in different food product samples as well as environmental samples. For example, Al-Ghamdi et al., confirmed the misuse of TCs antibiotics in poultry products in the eastern region of Saudi Arabia using a microbiological method^14^. Additionally, two other studies performed in the Al-Ahsa’a region have showed the presence of antibiotics residues in animals. The first study used LC–MS/MS to screen for nine antibiotics (quinolones, fluoroquinolones, sulfonamides and tetracyclines) residues in camel, cattle, and sheep tissues^15^. The second which was by Al-Nazawi et al., used the Delvotest P Multi plate test to screen for mainly TCs, streptomycin, and neomycin in dairy products^16^.

Much of the current literature utilizes liquid chromatography coupled with mass spectrometry LC–MS/MS as the most appropriate method for the detection and quantification of multiclass antibiotics in food and milk samples^17,18^. Other techniques have been reported for the determination of TCs antibiotics residues in milk samples includes capillary electrophoresis (CE-FL^19^ and CE-DAD^20^), a microbiological detection^21^ and a spectroscopic method^22^. Data from two recent review articles revealed the use of immunological kits as a monitoring tool for tetracyclines antibiotics residues in milk^12,17^. Table 1 summarizes some reported chromatographic methods for the determination of TCs antibiotics residues in milk samples by HPLC-DAD. These review articles pinpoint a number of similarities between the published methods. The vast majority of studies have utilized a C18 stationary phase with reverse-phase HPLC^12^. Additionally, it has been noted that binary mixtures of water-acetonitrile or water–methanol with different concentrations of organic components are more frequently used than tertiary mixtures (water-acetonitrile-methanol). Oxalic, formic, acetic and citric acids are among the most commonly used chemicals in the mobile phase^12,23^. Gradient elution systems have been reported with complex samples and antibiotics mixtures, making it possible to perform separation^12^.

**Table 1:** Determination methods of tetracycline antibiotics residues in milk samples by HPLC-DAD.

Compound(s)	Stationary phase	Mobile phase	Detection	Reference
TTR	C_18_ column	Isocratic elution: 0.01 M oxalic acid, ACN and MeOH (70:20:10 v/v/v)	HPLC-DAD at 385 and 276 nm	^29^
OXY, TTR _*The injection volume was 75 µL_	C_18_ column	Isocratic elution: 0.05 M potassium dihydrogen phosphate (pH 2.8) and ACN (80:20, v/v)	HPLC-UV at 232 nm	^26^
TTR, OXY, MNC, CTC, MTC and DC	C_18_ column	Gradient elution: ACN and NaH_2_PO_4_	HPLC-DAD at 350 nm	^30^
TTR, 4-epi TC and OXY	C_18_ column	Isocratic elution: 0.010 M Oxalic acid:ACN:MeOH (150:20:20 v/v/v)	HPLC-DAD at 365 and 280 nm	^31^
TTR, OXY, CTC and DC	C_18_ column	Gradient elution: 0.01 M Oxalic acid and ACN	HPLC-DAD at 360 nm	^32^
OXY, TTR, epi-CTC, CTC and DC	C_18_ column	Gradient elution: 0.01 M Oxalic acid and ACN	HPLC-DAD at 355 nm	^33^
OXY, TTR and CTC	C_18_ column	Isocratic elution: 0.01 M potassium dihydrogen phosphate:ACN:MeOH (70:20:10 v/v/v), pH 4	HPLC-DAD at 358 nm	This work

*OXY* Oxytetracycline, *TTR* tetracycline, *CTC* Chlortetracycline, *MTC* Methacycline, *MNC* Minocycline, *DC* Doxycycline, *4-epiTC* 4-epiTetracycline, *epi-CTC* epi-Chlortetracycline, *ACN* Acetonitrile, *MeOH* Methanol.

The main objective of the study was to determine tetracyclines antibiotics residues in milk samples from the Saudi market by means of HPLC coupled with the DAD technique.

## Experimental

### Materials and reagents

Reference materials of oxytetracycline (98.07% purity NMR), tetracycline (> 98.00%), chlortetracycline (> 95.00%) and the internal standard ornidazole (ORZ, > 99.00%) were purchased from Haoyuan ChemExpress Co., Ltd. (Shanghai, China). Potassium dihydrogen orthophosphate anhydrous (KH_2_PO4) was obtained from Loba Chemie Pvt. Ltd. (Mumbai, India). Ethylenediaminetetraacetic acid disodium salt dihydrate (Na_2_EDTA) was obtained from Sigma–Aldrich Chemie Gmbh (Steinheim, Germany). Orthophosphoric acid (H_3_PO_4_) was obtained from Avonchem Ltd. (Cheshire, UK). The acetonitrile and methanol solvents in the mobile phase were of UHPLC and HPLC grade. Deionized water was used in all experiments. Milk samples were purchased from multiple local markets and farms in the city of Riyadh. Tetracycline Rapid Immunoassay test kits were obtained from Meizheng Biotech Group, a PerkinElmer company (Beijing, China). The HPLC system (Waters, Milford, MA, USA) consisted of a Waters 1525 binary HPLC pump, a Waters 2998 photodiode array detector, and a Waters 2707 autosampler. The data were acquired and processed using Waters Empower 3 software.

### Chromatographic conditions

Chromatographic separations were carried out on a Reverse-Phase Macherey–Nagel C_18_ column (250 × 4.5 mm i.d., particle size 5 μm). The mobile phase was a mixture of 10 mM potassium dihydrogen orthophosphate: acetonitrile: methanol at a ratio of 70:20:10 (v/v/v), and the pH was adjusted to 4 with 0.01 M orthophosphoric acid. The mobile phase was filtered through a 0.45 µm Whatman filter paper followed by degassing for 10 min and then delivered at a flow rate of 1 mL/min. Analysis was performed at 25 °C, and the elution of compounds was monitored with a diode array detector (DAD) from 210 to 600 nm. The chromatograms were recorded at 358 nm, and the injection volume was 50 µl.

### Preparation of standard solutions and construction of calibration curves

Stock solutions (1) at concentrations of 1 mg/mL for OXY, TTR, CTC and internal standard ORZ were prepared in methanol. Further dilution was required to prepare mixed-stock solution (2) at a concentration of 10 µg/mL for each solution, and the working concentrations used were 0.09, 0.3, 0.5, 0.7, and 1 µg/mL. Internal standard stock solution (2) was prepared separately by the same procedure. The solutions were kept in a freezer (−20 ℃) and sealed from light for a period of one month^24,25^.

### Sample collection

Milk samples (n = 100) were categorized mainly according to their species into cow, camel, and goat milk. Other classifications included their source (local commercial product/imported commercial product/local farms), lifetime (fresh/long life), fat content (full fat, low fat and skimmed milk), and feeding (organic/nonorganic). Samples information are shown in Table 2. Milk was purchased from local Saudi markets and farms (Riyadh city) over months of October–November 2021 and February 2022.

**Table 2:** Milk sample information.

Milk category (total)	Source	Lifetime	Fat contents	Feeding
Cow milk (n = 78)	Commercial local products (n = 65) Commercial imported products (n = 10) Local farms (n = 3)	Fresh milk n = 25) Long life (n = 53)	Full fat (n = 61) Low fat (n = 15) Skimmed (n = 2)	Organic (n = 9) Nonorganic (n = 69)
Camel milk (n = 8)	Commercial local products (n = 2) Local farms (n = 6)	Fresh milk (n = 8 )	Full fat (n = 8)	Nonorganic (n = 8)
Goat milk (n = 14)	Commercial local products (n = 3) Local farms (n = 11)	Fresh milk (n = 14)	Full fat (n = 14)	Nonorganic (n = 14)

### Preparation of milk samples for analysis

Milk sample extraction involves adding an organic solvent to precipitate the protein and a chelating agent. This technique is common and has been utilized in many studies^23^. Samples were prepared by the following procedure. The extraction process began by mixing 2 mL of a milk sample with 0.4 mL of 0.2 M Na_2_EDTA and 0.6 mL of methanol in polypropylene centrifuge tubes. Once a homogenous solution had formed, the tube was centrifuged at 16,000 rpm for 20 min, filtered through a 0.22 µm Whatman syringe filter into an HPLC vial for analysis.

### Tetracyclines rapid immunoassay test

The Tetracycline Rapid Test Kit is a qualitative assay that determines the presence of tetracyclines antibiotics residues in cow’s and camel’s milk. The kit components should reach room temperature (20–25 ℃) before use. Milk samples were shaken and added to the microwells (200 µL of cow’s milk samples and 100 µL of camel’s milk samples diluted with 100 µL of deionized water). The coating conjugate powder was dissolved by pipetting the content up and down 5 times. The sample mixtures were incubated for 2 min at room temperature prior to putting the test strip into the microwells. The test strips were observed for color development for 5 min and then removed, and the results were interpreted within 1 min.

## Results and discussion

### Optimization of the chromatographic conditions

#### Effect of the organic modifiers and organic acid/inorganic salt

After reviewing the literature, it has been noted that many TCs analytical methods are carried out using oxalic acid as the organic acid solution in the mobile phase, in addition to acetonitrile and methanol as organic modifiers. Preliminary experiments on these conditions revealed the effects of each mobile phase component. For example, increasing the acetonitrile ratio above 20% significantly reduced the resolution. To optimize the method on reference materials, different concentrations of oxalic acid with different ratios of the organic modifier were studied. The preliminary mobile phase ratio was 70:20:10 (v/v/v) 10 mM oxalic acid solution, acetonitrile, and methanol, respectively. Consistent with previous publications, our results indicated that 25 mM oxalic acid showed optimum resolution over other tested concentrations (0, 10, 40, 50 and 60 mM). Our findings also depicted that shorter retention times were observed with 10 mM oxalic acid.

**Figure 1:**
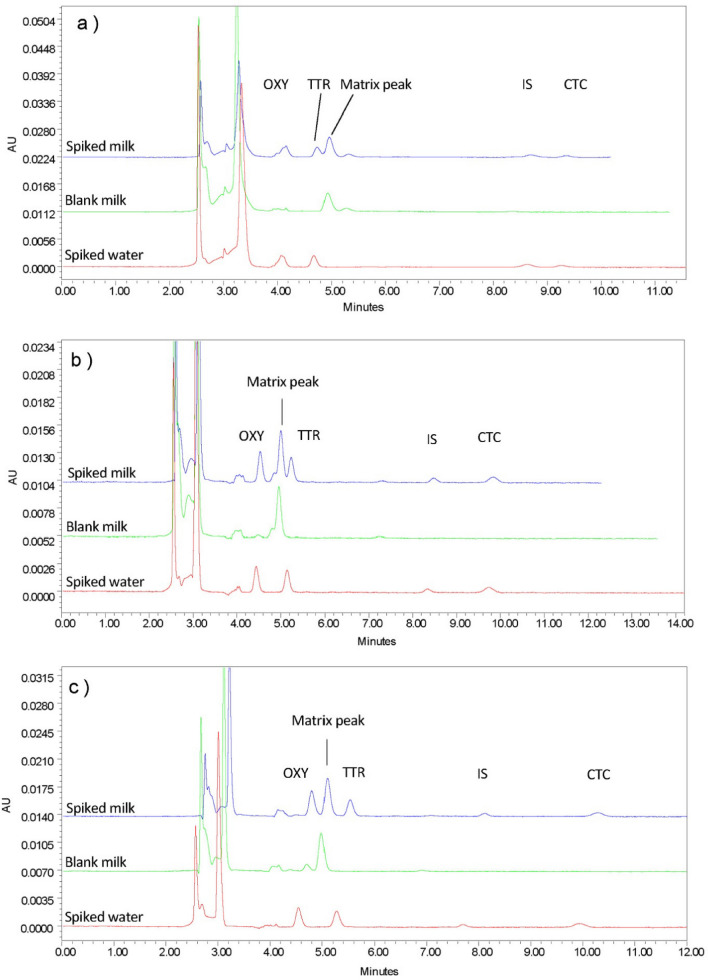
Effects of changing oxalic acid concentration on the drugs spiked cow milk. (**a**) 5 mM Oxalic acid:ACN:MeOH (70:20:10). (**b**) 10 mM Oxalic acid:ACN: MeOH (70:20:10). (**c**) 15 mM Oxalic acid:ACN:MeOH (70:20:10). Chromatographic conditions: Injection volume: 30 µL, Temperature: 25 ℃, Flow rate: 1 mL/min, Detection wavelength: 358 nm.

**Figure 2:**
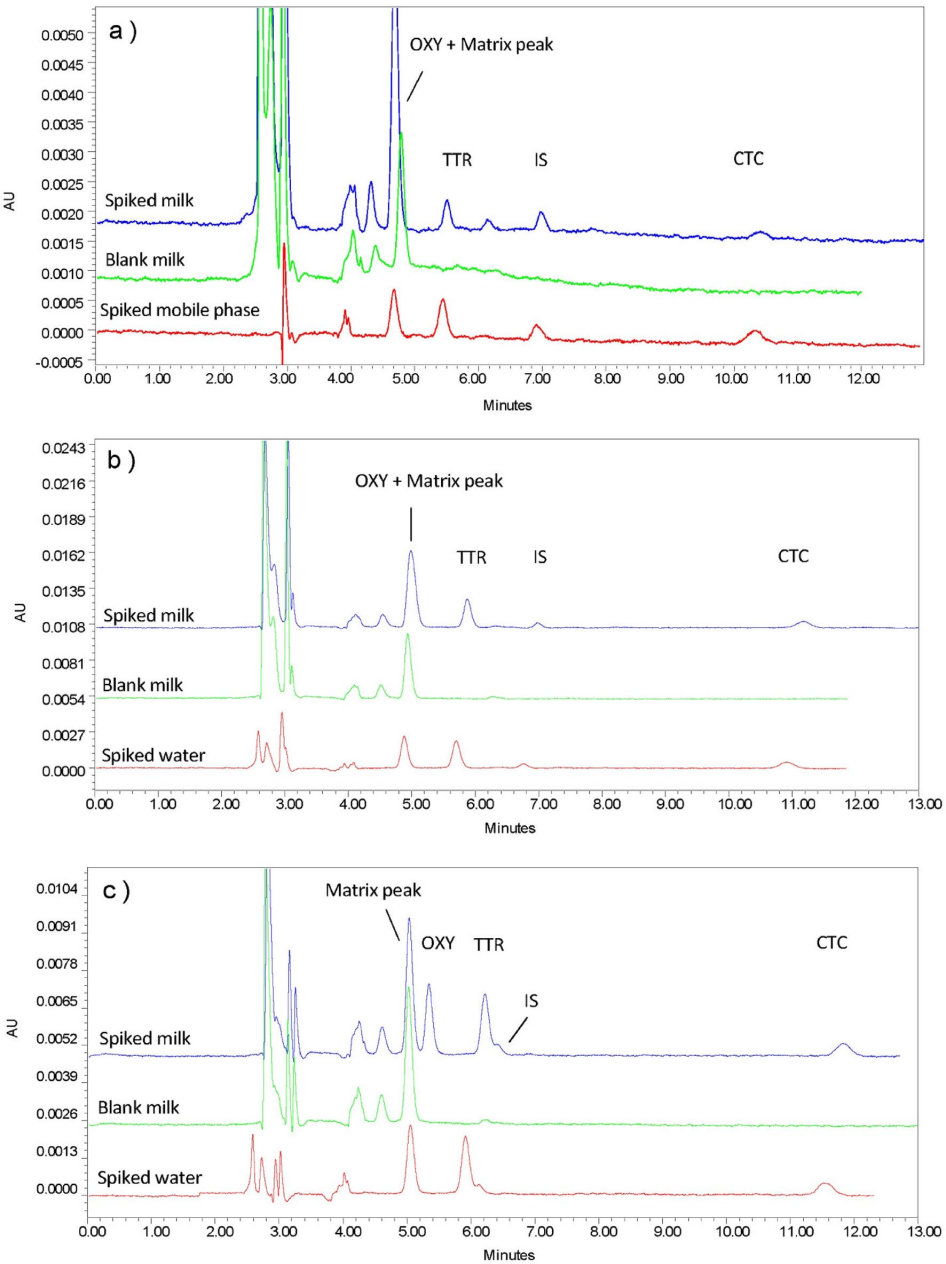
Effects of changing oxalic acid concentration on the drugs spiked cow milk. (**a**) 25 mM Oxalic acid:ACN: MeOH (70:20:10). (**b**) 30 mM Oxalic acid:ACN: MeOH (70:20:10). (**c**) 50 mM Oxalic acid:ACN: MeOH (70:20:10). Chromatographic conditions: Injection volume: 30 µL, Temperature: 25 ℃ ,Flow rate: 1 mL/min, Detection wavelength: 358 nm.

Regarding the organic modifiers, peak separation with satisfactory resolution was demonstrated with acetonitrile alone as the organic modifier at a ratio of 20%. However, using acetonitrile alone have increase the retention time to up to 30 min. which revealed that both methanol and acetonitrile are necessary for optimum conditions. Increasing ratios over 20% and 10% for acetonitrile and methanol, respectively resulted in a significant reduction in the resolution, whereas decreasing these ratios resulted in a longer run time. These findings broadly support the work of other studies in the case of analyzing the standard reference material, as this method has failed to provide an acceptable resolution when applied to milk matrices. Surprisingly, peak interference was demonstrated between a milk matrix component and TCs peaks. Therefore, no acceptable resolution was obtained, suggesting further method optimization on a spiked milk matrix. Figures 1 and 2 display an overview of the experimental chromatograms for the effects of changing the oxalic acid concentration on the drug-spiked milk matrix. Contrary to expectations, these trials were unable to achieve a reasonable resolution; methods failed to separate the matrix peak from the drug peaks, despite the different ratios and concentrations used. Overall, these findings indicate that the oxalic acid-based mobile phase is not suitable for milk matrices.

Previous work raised the possibility of replacing oxalic acid with potassium dihydrogen phosphate KH_2_PO_4_ as the inorganic salt in the mobile phase together with the organic modifiers for TCs antibiotics determination. For example, the determination of OXY and TTR in milk samples was carried out with using an isocratic elution system of 0.05 M potassium dihydrogen phosphate (pH 2.8)/ACN (80:20, v/v)^26^. Result of the preliminary practical experiment was promising, encouraging further research evaluating different KH_2_PO_4_ concentration with different ratios of organic modifier.

**Figure 3:**
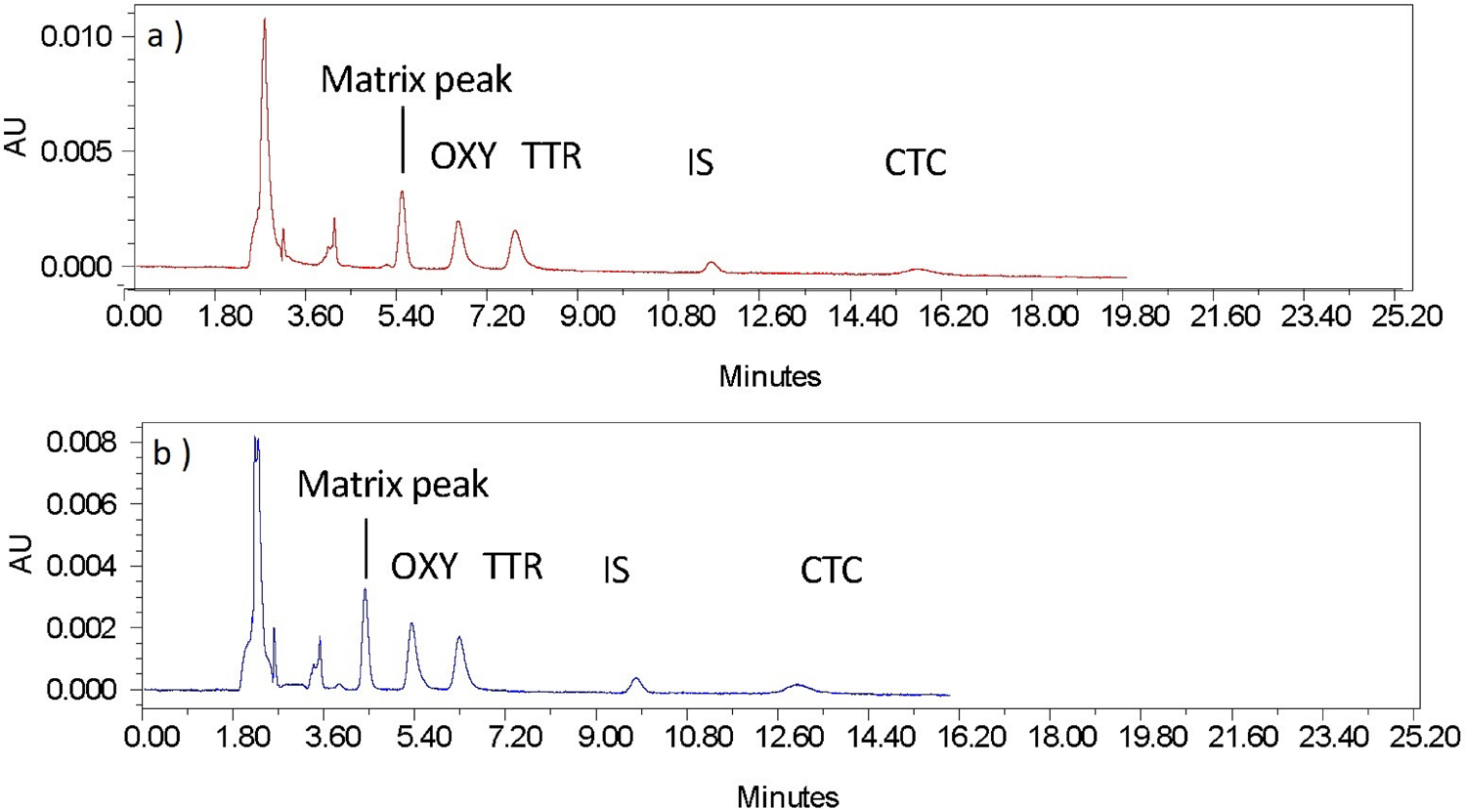
Effect of KH_2_PO_4_ concentration on spiked cow milk. (**a**) 25 mM KH_2_PO_4_: ACN: MeOH (70:20:10). (**b**) 10 mM KH_2_PO_4_: ACN: MeOH (70:20:10).

Initially, 0.025 M KH_2_PO_4_ with ACN and MeOH in different ratios were tested. However, decreasing KH_2_PO_4_ to 0.01 M showed a better resolution. Figure 3 represents the effect of changing the ratios of mobile compositions on the spiked milk matrix. Perhaps the most significant finding is that good separation is achieved with the mobile phase at a ratio of 70:20:10 of KH_2_PO_4_: ACN: MeOH. However, a reduction in the sensitivity was noted, which was expected to be due to the loss of the pharmacophore in the chemical structure of TCs and the pH of the mobile phase had changed. Hence, the pH of the mobile phase was evaluated to increase the sensitivity.

#### Effects of pH

**Figure 4:**
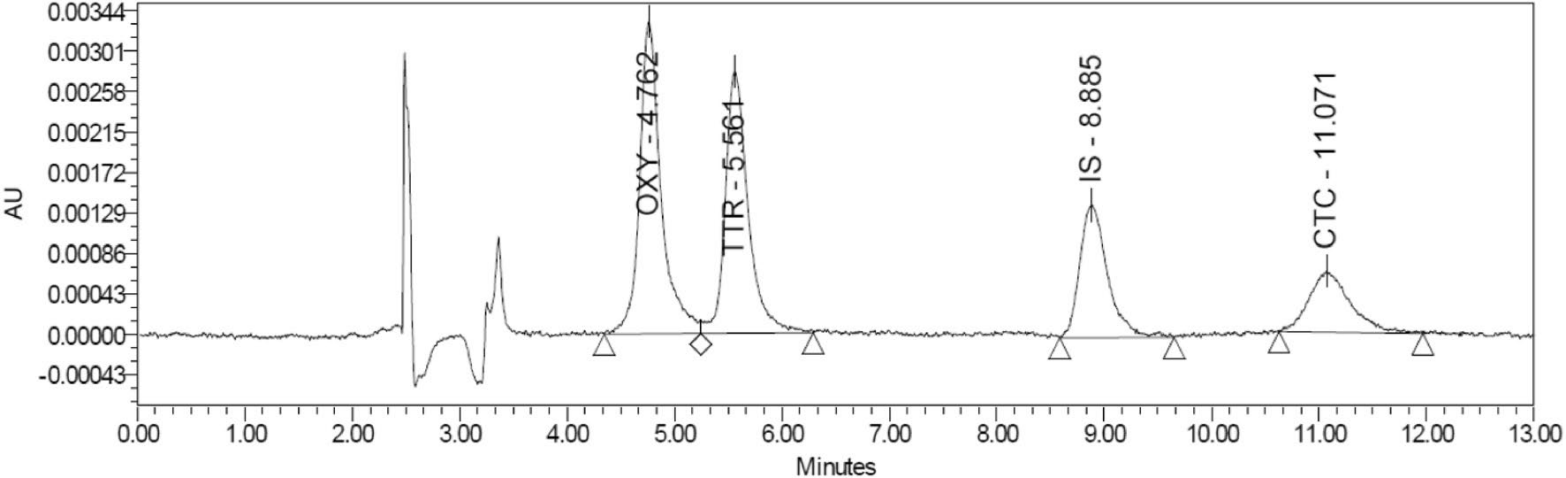
The chromatogram of the proposed method. Chromatographic conditions: 10 mM KH2PO4: ACN: MeOH (70:20:10), Injection volume: 50 µL, Temperature: 25 ℃, Flow rate: 1 mL/min, Detection wavelength: 358 nm.

The influence of pH was evaluated by using 0.1 M orthophosphoric acid to adjust the mobile phase mixture of 0.01 M KH_2_PO4:ACN:MeOH in a ratio of 70:20:10 in the pH range of 2–5.5. Changing the pH was evaluated in terms of the effect on resolution and the sensitivity. An increase in resolution was noted toward the pH of 5.5, while in this region sensitivity was lost. Decreasing the pH to near 2–3 improved the sensitivity, but resolution was diminished. At pH 4, the mobile phase showed a satisfactory resolution and an acceptable sensitivity for the purpose of the study, therefore, was chosen for the evaluation of milk samples. Figure 4 shows a chromatogram for the proposed method.

#### Effect of injection volume

Although only trace amounts of chlortetracycline (CTC) were detected, adjusting the pH of the mobile phase to 4 provided good resolution and sensitivity. Alternatively, increasing the injection volume to increase the detection and lower the quantification limits for CTC. A rule of thumb is to keep the injection volume as low as possible to avoid column overload and peak broadening^27^. A gradual increase in the injection volume was examined to ensure that low concentrations of CTC were detected without signs of peak broadening. The injection volume was set to 50 µL, as it is within the instrument capability, and no signs of peak broadening were observed (Fig. 4).

#### Selection of detection wavelength

**Figure 5:**
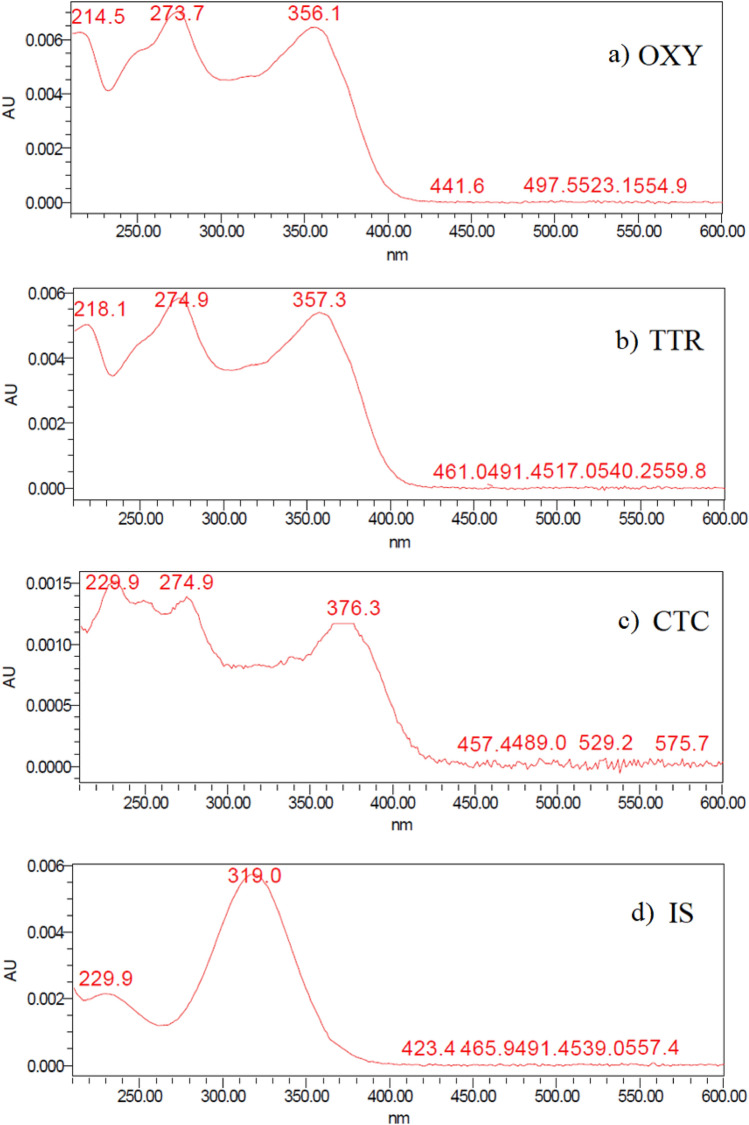
Absorbance spectra of the targeted analytes (**a**) Oxytetracycline, (**b**) Tetracycline, (**c**) Chlortetracycline and (**d**) Internal standard.

Absorption spectra of the three drugs, OXY, TTR, and CTC, were investigated with a DAD in the wavelength range of 200–600 nm. Spectra of the three drugs revealed two lambda max values, 267 nm and 358 nm. The wavelength of 358 nm was selected as the optimum detection wavelength since sample matrix is complex and many interfering peaks appeared at 267 nm. Figure 5 shows the absorbance spectra of the targeted analytes.

#### Optimization of the extraction method

The extraction procedure was developed to obtain a good recovery percentage while maintaining a concentrated sample. Previously reported methods indicated that food matrices has a high protein content, therefore, protein precipitation is employed in the pretreatment step^17^. Additionally, TCs molecules have a high affinity to form metal-complex chelates with polyvalent metal cations such as calcium and magnesium (Ca^+2^ and Mg^+2^), however, this complex formation could be prevented by adding a chelating agent to the sample^12^. Ethylene-diamine-tetra-acetic acid (EDTA) and organic solvents such as acetonitrile and methanol have been extensively used in the pretreatment of milk samples^23^. In the proposed extraction procedure, three key factors which could effect on the recovery percentage and the concentricity of the sample were identified: (1) the volume of the organic solvent, (2) the concentration of the chelating agent, and (3) the centrifugation time and speed. The methanol volume was kept to minimum to prevent sample dilution. Sodium EDTA (Na_2_-EDTA), which is more readily soluble in water than EDTA, was employed in this method as the chelating agent with no further adjustment of the pH. It was demonstrated that increasing centrifugation time and speed and the concentration of Na_2_-EDTA to 0.2 M resulted in a significant increase in the supernatant transparency and the percent recovery.

#### Method validation

Method validation was conducted by statistical analysis according to the guidelines of the International Council for Harmonization ICH^28^. Validation parameters were evaluated on the drug reference material and on the three different milk matrices (cow, camel, and goat). Selectivity and percent recovery were investigated on milk matrices in addition to linearity, limits of detection and quantitation, precision, and accuracy.

### Method validation on reference material

#### Linearity

The linearity response of each drug was determined by plotting the ratio of the drug peak area over the peak area of the IS versus the drug concentration, and then the regression equations were established. The calibration curve was linear in the range of 0.09–1 µg/mL (90–1000 ng/mL) for each drug. The correlation coefficients were > 0.9998 for each drug, showing that the method is linear in the specified range. The concentrations used for the calibration curve were 0.09, 0.3, 0.5, 0.7, and 1 µg/mL.

#### Detection and quantification limits

The detection and quantitation limits were determined according to the signal-to-noise ratio. The limit of detection was set as a signal-to-noise ratio of 3:1, while the limit of quantification was set as a signal-to-noise ratio of 10:1. OXY and TTR limits of detection were 20 ng/mL (0.020 µg/mL), while the detection limit of CTC was 80 ng/mL (0.080 µg/mL). The limit of quantification was 50 ng/mL (0.050 µg/mL) for OXY and TTR and 90 ng/mL (0.090 µg/mL) for CTC.

#### Accuracy and precision

Accuracy was evaluated by preparing different concentrations of drug mixtures within the linear range and a fixed concentration of Ornidazole as the IS (0.8 µg/mL) and then calculating % recovery and relative error. Table 3 shows a good % recovery (theoretical conc./practical conc. %) and small relative error for OXY, TTR, and CTC. For intraday precision, the same drug concentrations used to evaluate accuracy were analyzed three times on the same day and then on the next two days to evaluate the interday precision. The values of relative standard deviation (RSD) were calculated for each drug and concentration in Table 3. Low RSD values (< 2) indicate a high degree of precision.

**Table 3:** Intraday and interday precision and accuracy for the tetracycline drugs.

Compound	Standard concentration (µg/mL)	Mean % recovery ± SD	RSD (%)^a^	Er (%)^b^	Mean % recovery ± SD	RSD (%)^a^	Er (%)^b^
		Intraday precision and accuracy (n = 3)			Interday precision and accuracy (n = 9)		
OXY	0.1	97.90 ± 0.98	1.01	− 2.099	97.68 ± 1.12	1.15	− 2.32
	0.5	100.87 ± 2.08	0.41	0.87	99.72 ± 6.7	1.34	− 0.28
	0.8	100.21 ± 1.82	0.23	0.21	99.80 ± 2.79	0.35	− 0.20
TTR	0.1	100.41 ± 1.68	1.67	0.41	100.48 ± 1.88	1.87	0.48
	0.5	99.66 ± 4.01	0.80	− 0.34	98.89 ± 7.3	1.48	− 1.11
	0.8	99.52 ± 2.14	0.27	− 0.48	98.50 ± 6.91	0.88	− 1.50
CTC	0.1	99.10 ± 1.28	1.29	− 0.90	99.00 ± 1.27	1.29	− 1.00
	0.5	100.28 ± 1.32	0.26	0.28	99.64 ± 3.82	0.77	− 0.36
	0.8	99.95 ± 2.26	0.28	− 0.05	99.91 ± 2.37	0.30	− 0.09

^*a*^*RSD (%)* Percentage relative standard deviation.

^*b*^*Er (%)* Percentage relative error.

### Method validation on milk matrix

#### Selectivity

**Figure 6:**
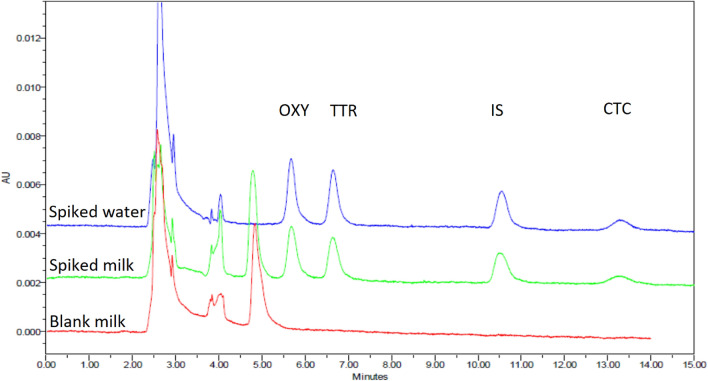
Selectivity of cow's milk matrix. mobile phase: 10 mM KH_2_PO_4_:ACN: MeOH (70:20:10), pH 4.

The selectivity of the method was assessed by examining the chromatograph of the blank milk sample (free from analytes tested by immunoassay and HPLC before spiking) against a spiked milk and water sample. All three milk matrices showed no interfering peaks at the retention times of the analytes; thus, the method was proven to be specific for TCs in the milk matrix. Figures 6 shows an example of the selectivity of the method on cow milk (the blank milk sample versus the spiked milk and water sample).

#### Extraction recovery percentages in the matrix

Recovery percentages were calculated as the ratio of the response from the spiked milk to the spiked water samples at the same concentration. The percentages recovery for OXY, TTR, and CTC in the milk matrix were within the specified range (80–120%). The mean recovery percentages ± SD of OXY, TTR and CTC in cowmilk were 94.45% ± 4.00, 88.77% ± 3.89 and 89.86% ± 2.41, respectively. For camel and goat milk, they were 101.20% ± 4.05, 98.43% ± 2.28 and 89.33% ± 11.03 and (96.45% ± 2.10, 92.73% ± 3.95 and 86.70% ± 1.95 for OXY, TTR and CTC, respectively.

#### Linearity

The linearity response of each drug in the three milk matrices was determined by plotting the ratio of the drug peak area over the peak area of IS versus the spiked drug concentration in the blank milk sample, and then the regression equations were established and presented in Table 4. The calibration curves were linear in the range of 0.09–1 µg/mL for OXY and TTR, whereas the range of CTC was 0.200–1 µg/mL (200–1000 ng/mL). Correlation coefficients were ≥ 0.9997 for each drug, showing that the method is linear in a specified range. Concentrations used for the calibration curve were 0.09, 0.2, 0.4, 0.6, 0.8 and 1 µg/mL.

**Table 4:** Regression equations and LOD/LOQ for the milk matrices.

Milk matrix	Linearity range (µg/mL)	Regression equation	*r*^2^	LOD (µg/mL)	LOQ (µg/mL)
**Cow milk**					
OXY	0.090 − 1	*y* = 0.0041*x* − 0.0063	0.9997	0.045	0.075
TTR	0.090 − 1	*y* = 0.0036*x* − 0.0258	0.9998	0.045	0.075
CTC	0.200 − 1	*y* = 0.0014*x* − 0.0140	0.9998	0.180	0.200
**Camel milk**					
OXY	0.090 − 1	*y* = 0.0043*x* − 0.0307	0.9998	0.050	0.080
TTR	0.090 − 1	*y* = 0.0036*x* − 0.0460	0.9999	0.050	0.080
CTC	0.200 − 1	*y* = 0.0012*x* − 0.0087	0.9997	0.180	0.200
**Goat milk**					
OXY	0.090 − 1	*y* = 0.0043*x* − 0.0472	0.9998	0.050	0.080
TTR	0.090 − 1	*y* = 0.0034*x* − 0.0392	0.9999	0.050	0.080
CTC	0.200 − 1	*y* = 0.0012*x* − 0.0224	0.9999	0.180	0.200

#### Limits of detection and quantification in milk matrix

The detection and quantitation limits were determined according to the signal-to-noise ratio. The limit of detection was set as a signal-to-noise ratio of 3:1, while the limit of quantification was set as a signal-to-noise ratio of 10:1. Table 4 shows the regression equations and r values for each drug in milk matrices in addition to the limit of detection (LOD) and limit if quantification (LOQ). Although the MRL of CTC in milk was 0.100 µg/mL, in here the most frequently detected concentration of CTC in the milk matrix was 0.180 µg/mL, and the quantification ability started at only 0.200 µg/mL, which is considered a drawback of the method.

#### Accuracy and precision

Precision and accuracy were investigated in spiked milk samples and analyzed three times on the same day and on the three successive days. Table 5 reveals that the proposed method has a high degree of accuracy and precision.

**Table 5:** Accuracy and precision of the method in each milk matrix.

Analyte	Spiked standard conc. (ng/mL)	Intraday precision and accuracy (n = 3)			Interday precision and accuracy (n = 9)		
		Mean % recovery ± SD	RSD (%)^a^	Er (%)^b^	Mean % recovery ± SD	RSD (%)^a^	Er (%)^b^
**Cow milk**							
OXY	0.25	97.69 ± 2.01	0.82	− 2.31	94.79 ± 9.65	4.07	− 5.21
	0.5	96.82 ± 5.78	1.19	− 3.18	98.08 ± 11.27	2.30	− 1.92
	0.7	101.01 ± 9.91	1.40	1.01	99.88 ± 14.45	2.07	− 0.12
TTR	0.25	97.58 ± 1.03	0.42	− 2.42	96.81 ± 5.6	2.31	− 3.19
	0.5	100.15 ± 8.39	1.68	0.15	99.18 ± 7.54	1.52	− 0.82
	0.7	100.44 ± 5.66	0.80	0.44	100.12 ± 10.66	1.52	0.12
CTC	0.25	96.84 ± 1.54	0.64	− 3.16	95.75 ± 4.11	1.72	− 4.25
	0.5	98.50 ± 1.59	0.32	− 1.50	97.3 ± 5.63	1.16	− 2.70
	0.7	101.13 ± 8.67	1.22	1.13	99.49 ± 10.95	1.57	− 0.51
**Camel milk**							
OXY	0.25	99.01 ± 3.16	1.27	− 0.99	99.12 ± 3.86	1.56	− 0.88
	0.5	101.37 ± 6.23	1.23	1.37	102.16 ± 6.17	1.21	2.16
	0.7	100.93 ± 5.59	0.79	0.93	101.78 ± 7.65	1.07	1.78
TTR	0.25	99.53 ± 1.14	0.46	− 0.47	96.7 ± 5.93	2.45	− 3.3
	0.5	100.97 ± 5.18	1.03	0.97	99.69 ± 8.98	1.8	− 0.31
	0.7	100.76 ± 3.84	0.54	0.76	98.79 ± 11.24	1.63	− 1.21
CTC	0.25	102.19 ± 2.36	0.92	2.19	100.04 ± 5.48	2.19	0.04
	0.5	101.87 ± 3.96	0.78	1.87	102.09 ± 6.64	1.3	2.09
	0.7	100.83 ± 2.22	0.32	0.83	100.14 ± 11.5	1.64	0.14
**Goat milk**							
OXY	0.25	100.85 ± 5.2	2.06	0.85	101.53 ± 3.78	1.49	1.53
	0.5	97.04 ± 5.63	1.16	− 2.96	97.91 ± 7.64	1.56	− 2.09
	0.7	98.65 ± 8.74	1.27	− 1.35	98.49 ± 8.5	1.23	− 1.51
TTR	0.25	101.91 ± 5.28	2.07	1.91	99.998 ± 7.28	2.91	− 0.002
	0.5	97.22 ± 3.29	0.68	− 2.78	96.69 ± 3.08	0.64	− 3.31
	0.7	99.5 ± 1.48	0.21	− 0.5	99.96 ± 3.11	0.44	− 0.04
CTC	0.25	98.87 ± 3.95	1.6	− 1.13	101.12 ± 7.04	2.78	1.12
	0.5	97.88 ± 4.92	1.005	− 2.12	98.97 ± 11.12	2.25	− 1.03
	0.7	98.77 ± 3.08	0.45	− 1.23	98.48 ± 4.65	0.67	− 1.52

^a^*RSD (%)* Percentage relative standard deviation.

^b^*Er (%)* Percentage relative error.

### Tetracyclines rapid test kit

The methodological approach taken in this study is a combination of qualitative and quantitative analysis. By employing a qualitative approach, it was easier to conduct this exploratory study for the purpose of scanning the highest possible sample size. The immunoassay technique is useful for identifying positive samples in a short time. The TCs rapid test kit used is a lateral flow assay that can qualitatively determines TCs residues in cow and camel milk (100 µg/kg). A test strip is composed of a sorbent pad on the lower end and two lines on a nitrocellulose membrane (T-line and C-line) is used. The T-line is the test line, which binds TCs molecules, whereas the C-line is the control line, which binds the secondary antibodies to indicate the validity of the test strip. The main component of the test is the gold-conjugated tetracycline antibodies that should be mixed with the sample before inserting the test strip. The kit is nonselective for each TCs antibiotics, and its sensitivity values (detection limits) was stated as 14 µg/kg for TTR and CTC and 10 µg/kg for OXY and Doxycycline.

**Figure 7:**
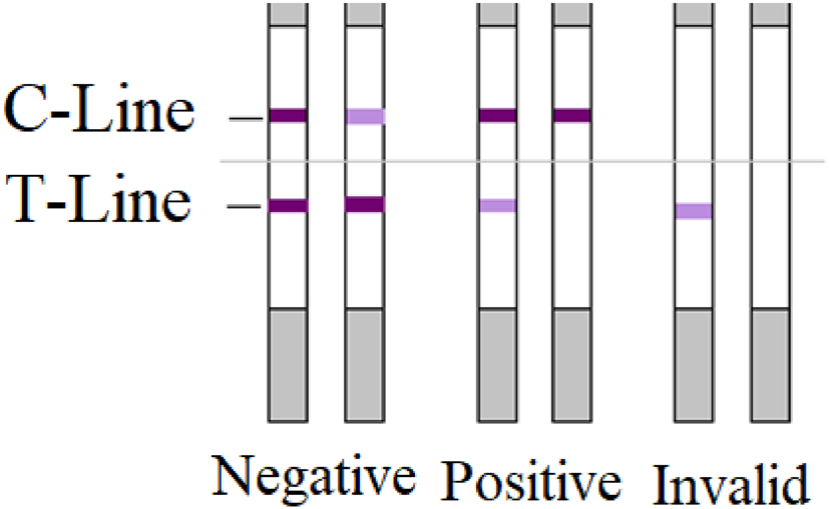
Negative sample (−): the sample is free of tetracyclines residues if the intensity of the T-line is greater than the C-line, and it is lower than limitation if their intensities are similar. Positive sample (+): TCs antibiotics residues are equal to the limitation if the intensity of the T-line is lighter than the C-line, and it is greater than limitation if only the C-line is visible. Invalid result: if the C-line is invisible.

Figure 7 shows the visual presentation of the test results for positive and negative samples and the invalid results. The interpretation of the result is based on visualizing and comparing the color intensities to determine if the TCs residues in the sample are greater than the MRL (100 µg/kg) or within the limitation, so there is a source of bias or uncertainty due to the self-reported nature of the result as an observational study. Thus, to minimize the false negative interpretation of the result, this kit was used to detect whether TCs antibiotics residues were present in a sample, regardless of level, so the sample that shows a near or equal degree of color similarity between the T-line and the C-line is considered a positive sample and thus categorized with the positive samples (only C-line visible or faint T-line) for further determination by HPLC-DAD analysis.

For quality control, a positive control (tetracycline: 14 ppm = 14 ng/mL) and a negative control sample were prepared according to the instructions and tested each time the kit was used, in addition to a milk sample spiked with 100 ng/mL TCs . This procedure ensures the sensitivity and validity of the kit during the storage time. In addition, one random sample of each of the five negative samples underwent analysis by HPLC-DAD to confirm the immunoassay result.

### Presence of OXY, TTR and CTC antibiotics residues in milk products

**Figure 8:**
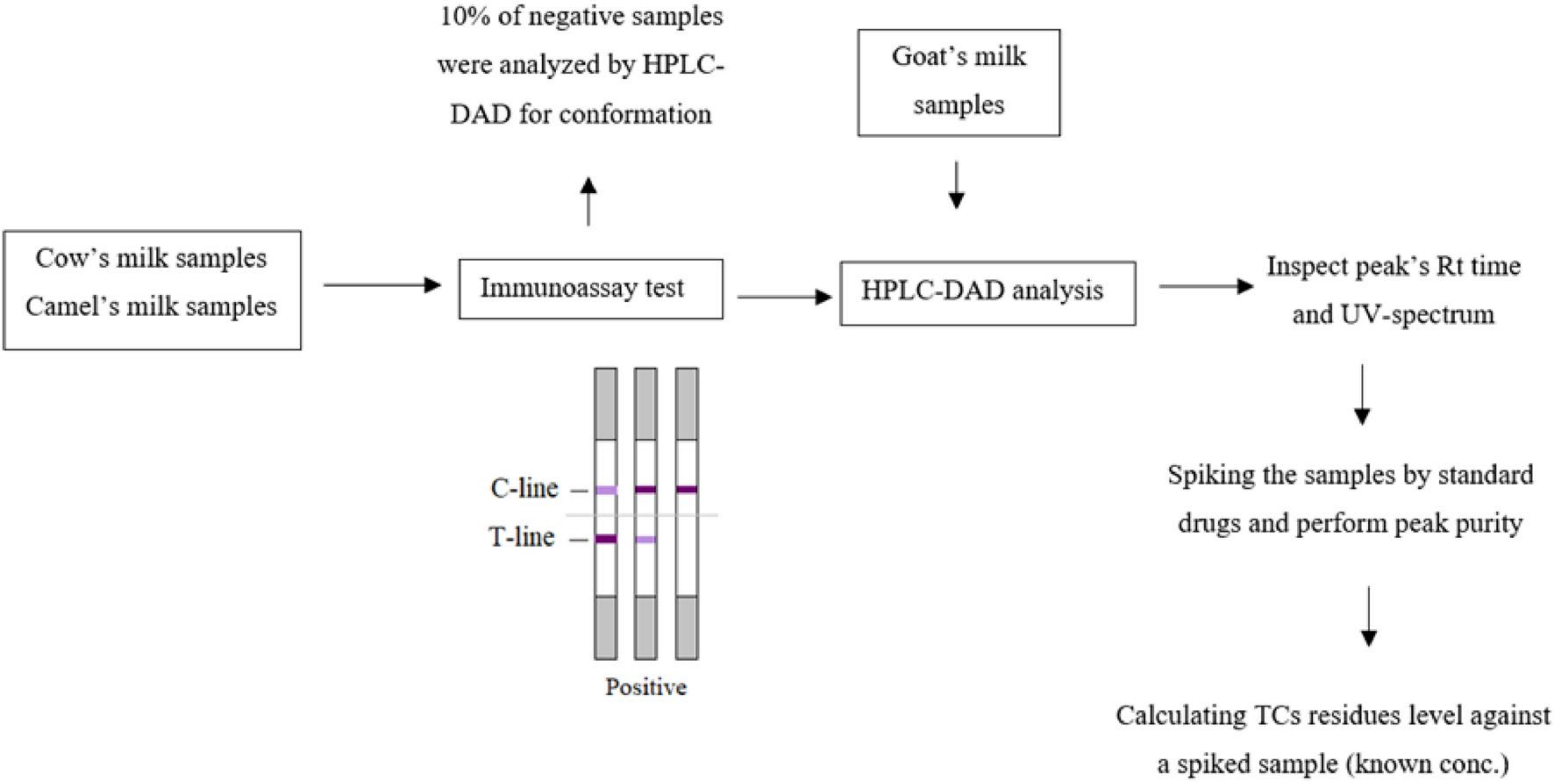
Schematic diagram of the workflow.

Cow and camel milk samples were first scanned for TC antibiotic residues by the immunoassay kit. Only samples that showed a dark T-line compared to the C-line with TCs antibiotics residues by the immunoassay kit were considered a negative sample and excluded from HPLC-DAD analysis. Samples showing a C-line only or a T-line that was faint or similar to the C-line in intensity were determined for TCs antibiotics residues by HPLC-DAD, together with the goat milk samples. On the day of analysis, spiked water and milk samples were prepared and injected to ensure the suitability of the chromatographic system. Milk samples were then prepared and injected into the HPLC-DAD system. Retention times of these peaks were compared to the spiked milk sample retention times and UV spectra. The following step was to spike these samples with a known concentration of the drugs and then inject them twice and examine their peak purity to exclude any matrix interference. The level of TCs antibiotics residues were determined by calculating their concentration against spiked milk with a known concentration of the drugs. The workflow is represented in Fig. 8.

For the qualitative test on cow and camel milk (there were 18 positive samples in two milk types; 17 samples were cow milk, and 1 sample was camel milk). These positive samples were further analyzed by the proposed method to determine TCs antibiotics residue levels.

As Table 6 shows, the occurrence of TCs residues in milk products (sample size = 100) was lower than the MRL (0.1 µg/ml). The prominent detected antibiotics were OXY and TTR with levels less than the MRL in most of the positive samples. Regarding total TCs antibiotics residues, only 9 samples (11.54%) exceeded the MRL. CTC was not detected in any of the samples, which is possibly due to the limit of detection of this method (0.180 µg/mL) which was higher than the MRL. However, this fact is not supported as the positive samples contain other TCs antibiotics and that the use of CTC is restricted.

**Table 6:** Presence of TCs residues in milk products (sample size n = 100).

Drug	Number of samples (n)	Cow milk		Camel milk		Goat milk
		78		8		14
OXY	Number of positive samples (% of total)	12	15.38%	−		−
	Mean of positive sample (µg/mL)	0.0533				
	Range of positive samples (µg/mL)	0.0225–0.090				
	Exceeds MRL of OXY (%)	0	0%			
TTR	Number of positive samples (% of total)	12	15.38%	1	12.5%	−
	Mean of positive sample (µg/mL)	0.0887		0.0390		
	Range of positive samples (µg/mL)	0.0265–0.1182				
	Exceeds MRL of TTR (%)	6	7.69%	0	0%	
CTC	Number of positive samples (% of total)	−		−		−
	Mean of positive sample (µg/mL)					
	Range of positive samples (µg/mL)					
	Exceeds MRL of CTC (%)					
	Samples exceeding the MRL by total TCs (%)	9	11.54%	0	0%	−

## Conclusion

TCs are commonly used in veterinary medicine as antibiotics or growth promoters. The inappropriate usage or lack of clear utilization protocols may lead to the presence of their residuals in livestock products. Our HPLC-DAD method shows an improved in resolution and sensitivity parameters which was validated according to the guidelines of the ICH. Recovery percentages are high (85–100%) with minimum matrix effects. Our finding indicates that in the majority milk samples obtained from Saudi market, TCs residues in are below MRL for TTR and OXY. The only exceptions were with 7.5% of the samples which exceed the MRL of TTR, and 11.5% of the samples which exceed the MRL for the sum of TTR and OXY, but not OXY alone. Our data reflects the good clinical practice of these two TCs in terms of usage in animal feeding and treatment in Saudi Arabia. Our method was not suitable for the determination of CTC in milk samples, as its quantification limit is two-fold the MRL .Thus, further optimization of the sensitivity is needed for the method to be able to determine CTC residues in milk samples. We recommend the concurrent use of other analytical techniques such as immunoassay in the routine monitoring of TCs as well as extending samples used to include milk from individual farms and non-commercials for closer investigation into the practice of withdrawal periods and treatment protocols.

## Supplementary Information

Supplementary Information.

## Data Availability

All data generated or analyzed during this study are included in this published article.
