# Supplementary material for: Qualitative immunoassay for the determination of tetracycline antibiotic residues in milk samples followed by a quantitative improved HPLC-DAD method
Source: Sci Rep. 2022 Aug 25;12:14502. doi: 10.1038/s41598-022-18886-2 (PMC9411586; doi:10.1038/s41598-022-18886-2)
Supplement: Supplementary file 1 — Supplementary Information. [file 41598_2022_18886_MOESM1_ESM.docx]

**Recovery data for cow’s milk :**

|  |  | **Spiked Milk** | | | **Spiked Water** | | |  | | |
| --- | --- | --- | --- | --- | --- | --- | --- | --- | --- | --- |
|  | **conc. ng/mL** | **Drug AUP** | **IS AUP 800 ng/ml** | **AUP Ratio** | **Drug AUP** | **IS AUP 800 ng/ml** | **AUP Ratio** | **Recovery %** | **SD** | **RSD** |
| **OXY** | 200 | 12822 | 25338 | 0.506038361 | 13415 | 26104 | 0.513905915 | 98.47% | 4.00 | 4.24% |
|  | 500 | 32642 | 26351 | 1.238738568 | 35396 | 26979 | 1.311983394 | 94.42% |  |  |
|  | 800 | 52385 | 28560 | 1.834208683 | 52719 | 26002 | 2.027497885 | 90.47% |  |  |
|  | | | | | | | **Average** | **94.45%** |  | |
| **TTR** | 200 | 10272 | 25338 | 0.405399005 | 12512 | 26104 | 0.479313515 | 84.58% | 3.89 | 4.39% |
|  | 500 | 28358 | 26351 | 1.076164092 | 31464 | 26979 | 1.166240409 | 92.28% |  |  |
|  | 800 | 46293 | 28560 | 1.620903361 | 47111 | 26002 | 1.811822168 | 89.46% |  |  |
|  | | | | | | | **Average** | **88.77%** |  | |
| **CTC** | 200 | 4296 | 25338 | 0.169547715 | 5069 | 26104 | 0.194184799 | 87.31% | 2.41 | 2.68% |
|  | 500 | 10571 | 26351 | 0.401161246 | 11752 | 26979 | 0.435598058 | 92.09% |  |  |
|  | 800 | 16895 | 28560 | 0.591561625 | 17057 | 26002 | 0.655988001 | 90.18% |  |  |
|  | | | | | | | **Average** | **89.86%** |  | |

Table 1: Recovery data and calculations for tetracyclines drugs in cow's milk matrix (injected in triplicate).

**Recovery data for camel’s milk :**

|  |  | **Spiked Milk** | | | **Spiked Water** | | |  | | |
| --- | --- | --- | --- | --- | --- | --- | --- | --- | --- | --- |
|  | **conc. ng/mL** | **Drug AUP** | **IS AUP 800 ng/ml** | **AUP Ratio** | **Drug AUP** | **IS AUP 800 ng/ml** | **AUP Ratio** | **Recovery %** | **SD** | **RSD** |
| **OXY** | 200 | 13693 | 14765 | 0.927395869 | 13494 | 14324 | 0.942055292 | 98.44% | 4.05 | 4.00% |
|  | 500 | 31000 | 14203 | 2.182637471 | 30135 | 14615 | 2.061922682 | 105.85% |  |  |
|  | 800 | 49868 | 14439 | 3.45370178 | 52024 | 14960 | 3.477540107 | 99.31% |  |  |
|  | | | | | | | **Average** | **101.20%** |  | |
| **TTR** | 200 | 11456 | 14765 | 0.775888927 | 11589 | 14324 | 0.809061715 | 95.90% | 2.28 | 2.31% |
|  | 500 | 25875 | 14203 | 1.821798212 | 26542 | 14615 | 1.816079371 | 100.31% |  |  |
|  | 800 | 41887 | 14439 | 2.900962671 | 43804 | 14960 | 2.928074866 | 99.07% |  |  |
|  | | | | | | | **Average** | **98.43%** |  | |
| **CTC** | 200 | 3759 | 14765 | 0.254588554 | 4383 | 14324 | 0.305989947 | 83.20% | 11.03 | 12.34% |
|  | 500 | 8578 | 14203 | 0.603956911 | 10670 | 14615 | 0.730071844 | 82.73% |  |  |
|  | 800 | 14133 | 14439 | 0.978807397 | 14348 | 14960 | 0.959090909 | 102.06% |  |  |
|  | | | | | | | **Average** | **89.33%** |  | |

Table 2: Recovery data and calculations for tetracyclines drugs in camel’s milk matrix (injected in triplicate).

**Recovery data for goat’s milk :**

|  |  | **Spiked Milk** | | | **Spiked Water** | | |  | | |
| --- | --- | --- | --- | --- | --- | --- | --- | --- | --- | --- |
|  | **conc. ng/mL** | **Drug AUP** | **IS AUP 800 ng/ml** | **AUP Ratio** | **Drug AUP** | **IS AUP 800 ng/ml** | **AUP Ratio** | **Recovery %** | **SD** | **RSD** |
| **OXY** | 200 | 12418 | 14107 | 0.880272205 | 13828 | 15069 | 0.917645497 | 95.93% | 2.10 | 2.18% |
|  | 500 | 31029 | 14297 | 2.170315451 | 32786 | 14919 | 2.197600375 | 98.76% |  |  |
|  | 800 | 51346 | 14739 | 3.483682746 | 53365 | 14500 | 3.680344828 | 94.66% |  |  |
|  | | | | | | | **Average** | **96.45%** |  | |
| **TTR** | 200 | 9610 | 14107 | 0.681222088 | 10663 | 15069 | 0.707611653 | 96.27% | 3.95 | 4.26% |
|  | 500 | 25070 | 14297 | 1.753514723 | 27992 | 14919 | 1.876265165 | 93.46% |  |  |
|  | 800 | 40177 | 14739 | 2.725897279 | 44674 | 14500 | 3.080965517 | 88.48% |  |  |
|  | | | | | | | **Average** | **92.73%** |  | |
| **CTC** | 200 | 3218 | 14107 | 0.228113702 | 3875 | 15069 | 0.257150441 | 88.71% | 1.95 | 2.25% |
|  | 500 | 8655 | 14297 | 0.605371756 | 10430 | 14919 | 0.699108519 | 86.59% |  |  |
|  | 800 | 14237 | 14739 | 0.965940702 | 16515 | 14500 | 1.138965517 | 84.81% |  |  |
|  | | | | | | | **Average** | **86.70%** |  | |

Table 3: Recovery data and calculations for tetracyclines drugs in goat’s milk matrix (injected in triplicate).
